# Quality control of *Platycodon grandiflorum* (Jacq.) A. DC. based on value chains and food chain analysis

**DOI:** 10.1038/s41598-023-41013-8

**Published:** 2023-08-28

**Authors:** Linlin Jiang, Hui Niu, Yuan Chen, Xing Li, Yulian Zhao, Chunhong Zhang, Minhui Li

**Affiliations:** 1 Inner Mongolia Hospital of Traditional Chinese Medicine, Hohhot, 010020 China; 2 Inner Mongolia Traditional Chinese & Mongolian Medical Research Institute, Hohhot, 010010 China; 3 Department of Pharmacy, Baotou Medical College, Baotou, 014040 China; 4 Inner Mongolia Key Laboratory of Characteristic Geoherbs Resources Protection and Utilization, Baotou, 014040 China

**Keywords:** Plant sciences, Environmental social sciences

## Abstract

*Platycodon grandiflorum* (Jacq.) A. DC. has been proposed as a medicine and food homology, thus playing an important role in disease prevention and health promotion, with great potential for research and value in clinical application. We aimed to analyze stakeholders' production behavior and financial performance from a value chain (VC) perspective and provide a basis for improving the quality of *P. grandiflorum* and the interests of stakeholders. *P. grandiflorum* collected from different producing areas were chemically analyzed, and the quality of platycodin D was evaluated. Rstudio3.6.0 was used to analyze the correlation between total platycodins (as platycodin D, platycoside E, and platycodin D_3_) and platycodin D in *P. grandiflorum*, providing the basis for quality control of *P. grandiflorum*. In addition, we studied the anti-inflammatory and anti-cancer activities of *P. grandiflorum* extract under different links. Based on the food chain energy pyramid, the transfer efficiency of active components of *P. grandiflorum* in different links was studied. Accordingly, 10 different types of VCs were determined in producing *P. grandiflorum*. Our results show that vertical coordination has led to a more consistent traceability system and strict regulation of supply chains.

## Introduction

*Platycodon grandiflorus* (Jacq.) A. DC. (Supplementary Fig. S1) is the only species of *Platycodon* A. DC. In the family of Campanulaceae. It is a perennial herb, and its dried roots have been used in Chinese medicine for thousands of years^1^. According to a record of traditional Chinese medicine dated 2000 years ago, *Platycodon grandiflorum* was mainly used as an expectorant for relieving sore throat and aiding in evacuating mucus. Later, it was documented in many other well-known medicinal works, including *Bencao Yanyi* (Song Dynasty, 1116 A.D.), *Bencao Jing Jizhu* (Liang Dynasty, 1565 A.D.), and *Bencao Gangmu* (Ming Dynasty, 1590 A.D.). Moreover, *P. grandiflorum* was used to cure chest congestion, diphtheria, and dyspnea, according to *Shang Han Lun* (Han Dynasty, 219 A.D.), a famous early Chinese document. According to *Bencao Yanyi* (Song Dynasty, 1116 A.D.), it was used to treat pulmonary abscesses. *P. grandiflorum* was also reported to be used for the treatments of mastitis, measles, dermatitis, and dysentery in *Wanbing Huichun* (Ming Dynasty, 1615 A.D.). Traditional Chinese medicine with *P. grandiflorum* as the main medicine has achieved good curative effects against respiratory diseases, such as acute upper respiratory tract infection, bronchitis, chronic bronchitis, bronchial asthma, pulmonary abscess, tuberculosis, and lung cancer^2^. Clinically, the best dose of *P. grandiflorum* is selected according to the disease, syndrome type, and symptoms, ranging from 3 to 50 g^3^*.* In addition, Chinese patent medicines containing *P. grandiflorum,* such as cough expectorant granules (Gansu Provincial Drug Standard, 1985), compound *P. grandiflorum* tablets (Jilin Provincial Drug Standard, 1986), Chuankening (Heilongjiang Provincial Drug Standard, 1986) are used to treat chronic bronchitis, cough, and other symptoms.

*P. grandiflorum*, as a medicine and food homology, is in great demand in the market. In North Korea, South Korea, Japan, and China (Yanbian region), *P. grandiflorum* is highly edible and is often used as food and processed into canned products, preserved fruit, kimchi, and other products^4^. *P. grandiflorus*’s young seedlings, roots, and flowers can be eaten, and they contain various nutrients (amino acids, trace elements, and unsaturated fatty acids)^5^. Moreover, *P. grandiflorum* mainly contains flavonoids, saponins, polysaccharides, fatty oils, fatty acids, and other chemical components^6^, of which triterpenoid saponins are the main active components^1^. Currently, the saponins isolated from *P. grandiflorum* include oleanane-type pentacyclic triterpenoid saponins. More than 80 kinds of triterpenoid saponins have been isolated^7^. It has been reported that platycodin A, platycodin D, and platycodin D_3_ are three compounds with high activity among *P. grandiflorum* saponins^8^. Among them, platycodin D is the iconic ingredient of *P. grandiflorum* and its processing and the index ingredient for the identification of *P. grandiflorum* medicinal materials and decoction pieces recorded in the 2020 edition of *Chinese Pharmacopoeia* (Part 1)^9^. Meanwhile, the flavonoids isolated and identified from *P. grandiflorum* are mainly dihydroflavones, flavones, and flavonoid glycosides. Additionally, *P. grandiflorum* contains a high content of fructose and inulin, and its root comprises 0.92% fat oil, with a high content of unsaturated compounds. The contents of linoleic and palmitic acids are higher at 63.24% and 29.51%, respectively^6^.

*P. grandiflorum* exhibits various pharmacological activities, such as relieving cough, anti-inflammatory, antibacterial, antioxidant, anti-tumor, lowering blood sugar levels, protecting the liver, and enhancing immunity^10–12^. *P. grandiflorum*-characteristic expectorant effect is closely related to the total platycodin content^13^. Platycodins and *P. grandiflorum* polysaccharides have anti-tumor effects^14^. Total platycodins and platycodin D have a higher scavenging ability against free radicals in vitro and a stronger activity than ascorbic acid at the same concentration, and they are concentration-dependent^15^. *P. grandiflorum* can prevent bronchitis and can effectively improve bronchial asthma symptoms. Arachidonic acid metabolism and glycerophospholipid metabolism are common regulatory pathways through which platycodin D exerts its antitussive and expectorant activities, and these metabolic pathways are closely related to anti-inflammation, immune-function regulation, and regulatory mechanisms^16^. Pretreatment of Lipopolysaccharides (LPS)-stimulated RAW264.7 cells with *P. grandiflorum* extract significantly reduced the levels of proinflammatory cytokines, such as TNF-α, IL-6, and IL-1β, and inducible nitric oxide synthase (iNOS). In addition, fermented *P. grandiflorum* extracts relieved airway inflammation and cough reflex sensitivity^17^. Primary bronchial lung cancer is the most common primary malignant lung tumor. According to the 2018 Global Cancer Statistics report, the incidence of lung cancer remains the highest among all cancers, accounting for approximately 11.6%). *P. grandiflorum* is an effective medicine for treating lung diseases and has been proven to have a therapeutic effect in treating malignant tumors^18^. The applications of *P. grandiflorum* in food, cosmetics, medicine, and healthcare products are detailed in Supplementary Table S1.

Value chain (VC) analysis has been applied to various consumer goods; however, only a few studies have investigated the VCs of medicinal plants and their derivatives^19^. The concept of VC generally describes all processes, from the raw material to the final product, via different parties^20^. The focus of VC research has been two-fold: understanding how different types of VCs bring competitive advantage by changing how products are processed or sold and evaluating the socio-economic benefits, disadvantages, and risks of the different members in the chain^21, 22^. However, at present, the development of the traditional Chinese medicine industry is not balanced; the synergistic effect of industrial clusters is weak, the capacity for product output is not high, the market share is small, and there exists information asymmetry owing to the difficulty in quality control of traditional Chinese medicine products^23^. Moreover, there is a lack of quality control studies on the cultivation and trading of *P. grandiflorum*. The industry has a pattern of “small size and dispersity”; thus, standardized and intensive development has yet to be established. This affects the output of *P. grandiflorum* products and consequently limits the economic income of farmers and other stakeholders.

The food chain underlies the relationship in which organisms in the ecosystem depend on food and represents one of the cornerstones of ecology. Since 1927, when it was proposed by Elton, it has become one of the most widely studied concepts in empirical and theoretical ecology^24^. Food chains are characterized by interconnected structural, functional, and dynamical properties^24^. Nutrients in food are easily quantifiable and flow through the food chain system. The direction of nutrient flow not only directly affects the productivity of an agricultural system but is also related to the efficiency of agricultural resource utilization, environmental quality, and human health^25^. Therefore, further studies are needed to investigate the utilization efficiency along the food chain of *P. grandiflorum*.

The quality of plant products is influenced by many external factors, including various inspections along the product VC to improve the quality and safety of cultivated medicinal plants, resulting in dynamic quality control^13^. The VC can be used to improve overall product quality and traceability through an interdisciplinary research approach. This paper focused on *P. grandiflorum* as the research object and studied its food chain and VC to address the following issues. First, the quality of *P. grandiflorum* and the behavior of stakeholders were investigated from the perspective of capital risk, and the different capital risks of *P. grandiflorum* were compared to explore a potential quality control strategy. Second, according to the energy transfer in the food chain, the active ingredients and anti-cancer and anti-inflammatory activities of *P. grandiflorum* were used as energy indicators to compare the biological activity and active components transfer efficiency of different links in the VC of *P. grandiflorum* in Chifeng, Inner Mongolia, to provide a basis for ensuring and improving its quality.

## Materials and methods

### VC analysis

#### Fieldwork

The harvesting period of *P. grandiflorum* was divided into spring and autumn. Based on literature research, the origin survey of *P. grandiflorum* was carried out in September 2020, and several investigations were carried out in Inner Mongolia, Shanxi, Hebei, and Anhui during the cultivation and harvest periods, covering the main producing areas and medicinal materials trading centers of *P. grandiflorum*. This study randomly interviewed farmers, wholesalers, retailers, and persons in charge of firms in major growing areas or trading centers to collect information regarding *P. grandiflorum* origin and quality^20^. The survey information included basic information on the respondents (education level and work and training experience); production process of *P. grandiflorum*; planting, processing, storage, and harvesting methods of *P. grandiflorum*; output of *P. grandiflorum*; sales volume of *P. grandiflorum*; sales channels of *P. grandiflorum*; specifications and grades of *P. grandiflorum*; methods of quality control; quality monitoring; factors affecting the quality and sales of *P. grandiflorum*; and stakeholders’ labor and non-labor costs. In addition, respondents were encouraged to provide any information they wanted. The interviews were recorded using a tape recorder and preserved by the first author. The questionnaires are Supplementary Table S2. We also researched two representative medicinal materials markets, namely the Bozhou and the Anguo Chinese medicinal materials market. Furthermore, we conducted telephone interviews to collect information from the retailers of the Chengdu Lotus Pond Chinese herbal medicine market.

By investigating the different links in the production process of *P. grandiflorum*, we drew different VCs of *P. grandiflorum*. First, we interviewed farmers, wholesalers, and retailers using a snowball sampling method, starting from the planting of *P. grandiflorum*, along the production chain, until the marketing stage, to identify the main links and stakeholders related to the production, source, and direction of *P. grandiflorum* and quality problems that may occur in different links. Second, the interview information on the *P. grandiflorum* industry was summarized and integrated. Next, the main production activities were linked to the stakeholders, and a VC of *P. grandiflorum* was drawn. A total of 48 *P. grandiflorum* samples were collected from various production areas and stakeholders. The content of platycodin D, combined with the risk situation during the investigation, was used as the quality standard for evaluating *P. grandiflorum* samples. Ultimately, we compared the advantages and disadvantages of different VCs and discussed the relationship between financial producer behavior and the quality of different VCs.

#### *P. grandiflorum* samples

A total of 48 *P. grandiflorum* samples were collected from nine provinces in China (Supplementary Table S3, Fig. S2), some of which were collected, and some were purchased. All samples we collected were artificially planted *P. grandiflorum*, and we obtained permission to collect *P. grandiflorum*, which complies with the IUCN Policy Statement on Research Involving Species at Risk of Extinction and the Convention on the Trade in Endangered Species of Wild Fauna and Flora (statement from “Related files”). To maintain the sample quality within an acceptable range, the samples for content determination analysis were only collected from biennial samples. All *P. grandiflorum* samples used in the study were identified by Prof. Minhui Li of Baotou Medical College. A voucher specimen was deposited in the Herbarium of Baotou Medical College (Specimen No.: 150428180624008LY) for future reference.

#### Reagents and chemicals

A high-performance liquid chromatography (HPLC) system was purchased from Thermo Fisher Scientific “Ultimate 3000, Waltham, MA, USA”. A pulverizer “ST-08, 1800 W, China”, laboratory water purifier “AMFI-5–P, Yiyang Enterprise, China”, electronic analytical balance “AR153O, Mettler Toledo, Columbus, OH, USA” and 1260 Evaporative Light Scattering Detector “Agilent, Santa Clara, CA, USA” were also acquired. The methanol and acetonitrile used were HPLC grade, while all other reagents were of analytical grade. The reference compounds platycodin D_3_, platycodin D, and platycoside E were purchased from Chengdu Pufei De Biotech Co., Ltd (Sichuan, China).

The reagents used to analyze heavy metal pollutants were HNO_3_ and HClO_4_ at suprapure-grade. Other reagents were of analytical reagent grade unless otherwise stated. The element standard solutions used for the assay of the contents of Pb, Cd, As, Hg, and Cu were supplied by the National Institute of Metrology (Beijing, China).

All analytical standards of the studied pesticides were of high purity and certified upon purchase from the Agro-Environmental Protection Institute, Ministry of Agriculture and Rural Affairs (Beijing, China) as having a purity higher than 99%. The solvents, acetonitrile (ACN), acetone, methanol, and n-hexane, were of HPLC grade “Thermo Fisher Scientific, United States.” Magnesium sulfate (MgSO_4_) and sodium chloride (NaCl) were obtained from Aladdin (China), with a purity exceeding 99%. Other reagents were of analytical reagent grade unless otherwise stated.

#### Main chemical indexes in *P. grandiflorum*

All analyses were performed on a “Thermo Ultimate 3000” system. Gradient elution was conducted with (A) acetonitrile and (B) water as mobile phases. Platycodin D was detected under the following conditions. A “Hypersil GOLD C18 Column” (250 mm × 4.6 mm, 5 μm) was used for chromatography at 30 °C. The gradient was as follows: 0–15 min, 15–25% A; 15–35 min, 25–27% A; and 35–50 min, 27–30% A. The flow rate was 1.0 mL/min, and the sample injection volume was 10 μL. Conditions for determining platycoside E and platycodin D_3_ were as follows. Sample analysis was carried out on a C18 column at a 1.0 mL/min flow rate, with the gradient at 0–30 min, 15–30% A and 30–50 min, 30–100% A, and a column temperature of 30 °C. HPLC-ELSD detected three saponins with a drift tube temperature of 109 °C and an N_2_ carrier gas flow rate of 3.0 L/min.

#### Detection of heavy metal contaminants

The Pb, Cd, As, Hg and Cu amounts in RA were analyzed using atomic absorption spectrometry^9^. A “Thermo ICE 3000” atomic absorption spectrometer with a deuterium background corrector was used. Plant samples' Pb and Cd contents were determined using an HGA graphite furnace with argon as the inert gas. Cu was assayed out in an air-acetylene flame. Other measurements were based on cyanide complex processing^9, 22, 26^.

#### Detection of pesticide residues

The organochlorine residues, including total BHC (α-BHC, β-BHC, γ-BHC, and δ-BHC), DDT (pp’-DDE, pp’-DDD, op’-DDT, pp’-DDT), and pentachloronitrobenzene (PCNB), were determined using gas chromatography-tandem mass spectrometry^9^ “Thermo TRACE 1300 gas chromatograph” with a DB-5MS capillary column (0.25 mm × 30 m × 0.25 µm). Nitrogen was used as the carrier gas with a purity of 99.99%. A full auto-tune of the mass spectrometer was performed before analysis. The transfer-line temperature was set to 300 °C, the manifold temperature 50 °C, and the ion-trap temperature 250 °C. The flow rate was 1.0 mL/min, and the sample injection volume was 1 µL.

#### Platycodin correlations

Correlation analysis of different platycodins was carried out to provide a basis for further quality control. The content data of platycodin D, platycoside E, and platycodin D_3_ were extracted from the literature and uniformly adjusted to percentage content, that is, the percentage of each chemical component in the dry weight of the entire medicinal material. Rstudio3.6.0 was used to analyze the correlation between total platycodins and platycodin D, providing a basis for the quality control of *P. grandiflorum*. Representative samples were randomly selected from Chifeng, Inner Mongolia, to determine platycodin D, platycoside E, and platycodin D_3_ levels. Rstudio version 3.6.0 was also used to analyze the correlation between total platycodins and platycodin D in *P. grandiflorum* samples from Chifeng.

### Food chain analysis

*P. grandiflorum* has three common circulation forms in VC: freeze-dried medicinal materials (fresh samples of *P. grandiflorum* were collected and directly freeze-dried to maintain the freshness of the medicinal materials and facilitate comparison between fresh *P. grandiflorum* and primary processed *P. grandiflorum* from the origin), original medicinal materials (*P. grandiflorum* after primary processing at the origin), and prepared drug in pieces. These products correspond to the harvest of *P. grandiflorum*, the initial transport link, and the sales link to hospitals. Samples of *P. grandiflorum* were collected again in Chifeng, Inner Mongolia, including fresh raw *P. grandiflorum*, original medicinal materials, and prepared drug in pieces (Supplementary Fig. S3). Then we analyzed the food chain of the *P. grandiflorum* from Chifeng. The anti-cancer and anti-inflammatory activities and content of platycodin D of *P. grandiflorum* samples are similar to the definition of energy in the food chain. Consequently, the “energy” changes during harvesting, transport, and selling of the *P. grandiflorum* were analyzed.

####  Anti-cancer and anti-inflammatory activity of *P. grandiflorum* extract using different links

##### Plant extract

The roots of *P. grandiflorum* from the Chifeng area of Inner Mongolia were selected to analyze the anti-cancer and anti-inflammatory activities of freeze-dried medicinal materials, original medicinal materials, and prepared drugs in pieces. Briefly, 10.0 g of powdered *P. grandiflorum* medicinal materials, prepared drug in pieces sample, and freeze-dried medicinal materials sample were collected and extracted using 150 mL 75% ethanol at reflux for 3 h. After repeating the process twice, the filtrate was combined, concentrated, and freeze-dried to obtain the plant alcohol extract.

##### The effect of *P. grandiflorum* extract on A549 cell viability was detected using a CCK-8 kit

A549 cells were purchased from “Shanghai Jinyuan Biotechnology Co., Ltd.” (Shanghai, China) and were cultured in Dulbecco's Modified Eagle Medium (DMEM). A549 cells at the logarithmic growth stage were digested with 0.25% trypsin, and cells were counted and inoculated on 96-well plates at a density of 5 × 10^3^/well for 24 h. The treatment group was supplemented with *P. grandiflorum* extract (25, 50, 100, and 200 μg/mL), and 1 μg/mL LPS was added to each well for 24 h.

After 24 h, 10 µL CCK-8 solution was added to each well, and the cells were cultured in an incubator for another 4 h. Cell viability [Eq. (1)] and IC50 were evaluated by measuring the absorbance at 450 nm using a microplate reader^27^ “Tecan, Infinite, CH”.

1 $${\text{Cell survival rate}}\,\left( \% \right)\,= \,\left( {{\text{OD}}_{{{\text{experimental group}}}} - {\text{OD}}_{{{\text{blank group}}}} } \right)\,/\,\left( {{\text{ OD}}_{{{\text{control group}}}} - {\text{OD}}_{{{\text{blank group}}}} } \right)\, \times \,{1}00\% .$$

##### The effect of *P. grandiflorum* extract on RAW264.7 cell viability was detected using a CCK-8 kit

A total of 38.8, 76.5, and 86.3 mg of the original medicinal materials, prepared drug in pieces, and freeze-dried medicinal materials of *P. grandiflorum*, respectively, were placed in a 1.5 mL centrifuge tube. Next, 97, 191.25, and 215.75 μL DMSO were added and mixed evenly to form 400 mg/mL high-concentration reserve solutions. Finally, the extracts were diluted to 400 mg/mL, 200 mg/mL, 100 mg/mL, 50 mg/mL, and 25 mg/mL. In the subsequent experiment, serum-free DMEM was used to dilute each concentration to a final concentration of 25 μg/mL, 50 μg/mL, 100 μg/mL, 200 μg/mL, and 400 μg/mL, respectively.

RAW264.7 cells at the logarithmic growth stage were digested using 0.25% trypsin, and the cells were counted and inoculated on 96-well plates at a 5 × 10^5^/well density. After culturing the cells in 96-well plates for 24 h, the treatment group was supplemented with different concentrations of *P. grandiflorum* extract (25, 50, 100, 200, and 400 μg/mL), and 1 μg/mL of LPS was added to each well for 24 h^28^.

After 24 h, 10 µL CCK-8 solution was added to each well, and the cells were cultured in an incubator for another 4 h. Cell viability [Eq. (1)] and IC50 were evaluated by measuring the absorbance at 450 nm with a microplate reader.

##### Statistical analysis

Experimental data were processed and analyzed using GraphPad Prism 8 software (GraphPad Software, San Diego, CA, USA). We used a single-factor variance test for statistical analysis. Data were presented as the mean ± SD. Experiments were performed in triplicate, and statistical significance was set at P < 0.05 was considered statistically significant.

#### Transfer efficiency of active components in the food chain of *P. grandiflorum* for each nutrient level

The energy transfer efficiency of the food chain is characterized by a step-by-step energy decrease as it flows along the food chain^29^. This can be represented by an energy pyramid as follows:

2 $$ {\text{Energy transfer efficiency }}\left( \% \right) \,= \,{\text{ assimilation of the previous trophic level}}/{\text{assimilation of the next trophic level }} \times { 1}00\% . $$

Food chain energy transfer efficiency refers to the percentage of energy in one level of the food chain that can be consumed by the next level. The trophic level refers to any of the layers of an ecosystem with the same position in the food chain. Food energy circulation in the ecosystem is divided into different levels according to the location of the food chain. Assimilative amount refers to the total chemical energy obtained by a trophic level from the external environment. It is represented by the respiratory consumption of this trophic level, the energy flow from this trophic level to the next trophic level, the energy flow from this trophic level to thedecomposer, and the unused energy.

Therefore, based on the food chain energy pyramid, we explored the transfer efficiency of active components at different links. We calculated the content of platycodin D (Supplementary Table S4) in freeze-dried *P. grandiflorum* without peeling, freeze-dried *P. grandiflorum* with peeling, original medicinal materials, and prepared drug in pieces in the Chifeng area of Inner Mongolia, which respectively correspond to the four progressive nutrient levels of *P. grandiflorum* food chain. There are 3 samples of *P. grandiflorum* samples of the same nutrient level, and the average values of the three samples were used for calculation. Equation (3), which is transformed from the above formula, was used for the calculation.

3 $${\text{Active components transfer efficiency }}\left( \% \right) \,= {\text{content of the nutrient level/content of the total nutrient level}}\,\times \,{1}00\% .$$

The transfer efficiency of active components in the food chain of *P. grandiflorum* was the ratio of the content of platycodin D in the current nutritional grade to that in all nutritional levels. "content of the nutrient level" is the mean of the platycodin D content of *P. grandiflorum* at current nutrient level, and" content of the total nutrient level" is the sum of the 4 nutrient levels.

## Results

### Quality control of *P. grandiflorum*

**Figure 1:**
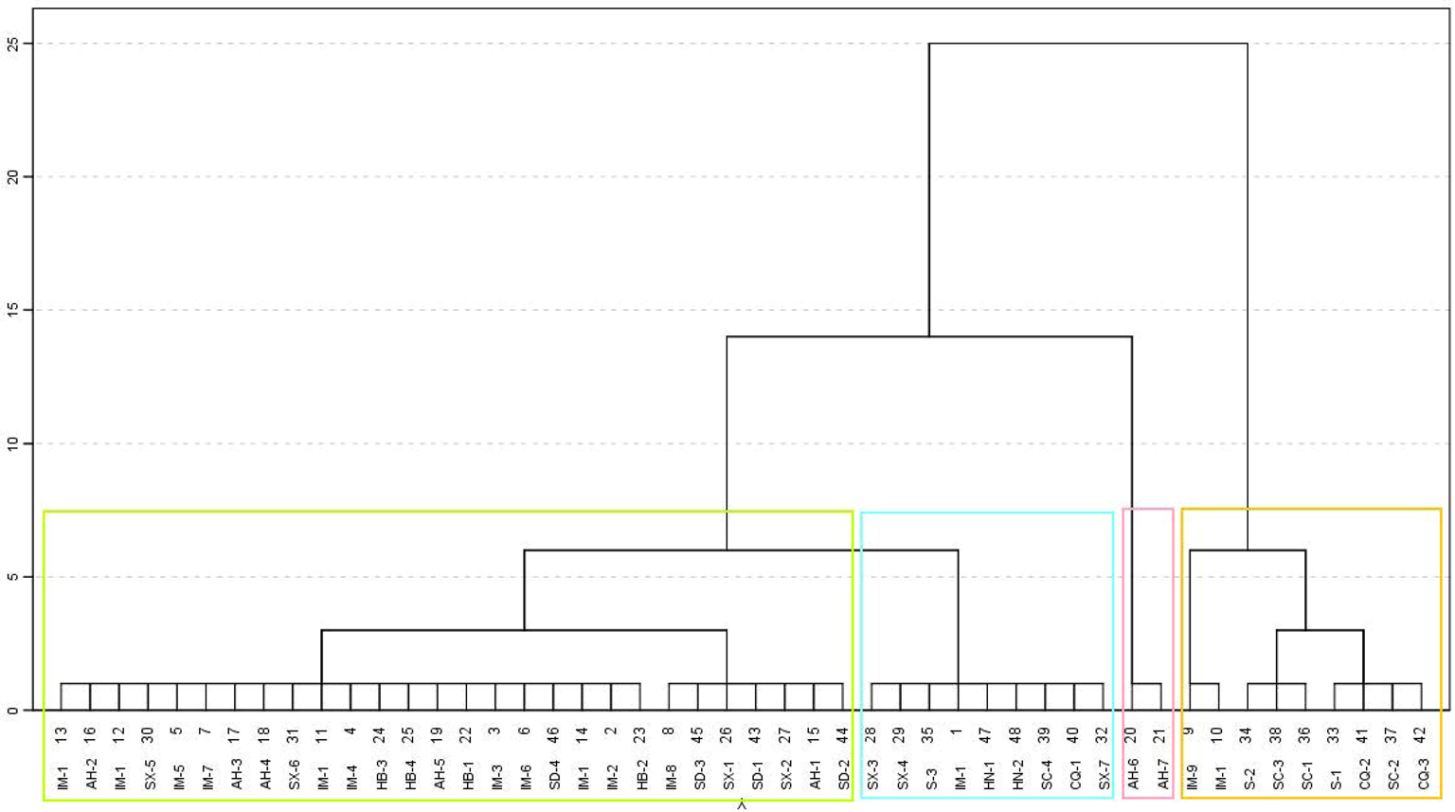
The cluster diagram of platycodin D from different producing areas (IM: Inner Mongolia, AH: Anhui, HB: Hebei, SX: Shaanxi, S: Shanxi, SC: Sichuan, CQ: Chongqing, SD: Shandong, HN: Henan) (Based on the content of platycodin D as the index, the colored rectangle indicates that the content of platycodin D in these regions is clustered into one class, and different colors represent different clusters).

A total of 48 samples were collected during the VC investigation, and the platycodin D content results are summarized in Supplementary Table S5. In addition, we used the cluster analysis method of SPSS to analyze platycodin D content in 48 samples collected from different producing areas (Fig. 1). The platycodin D content of *P. grandiflorum* from different producing areas could be roughly divided into 4 clusters. The Shanxi, Sichuan, Chongqing, and Tongliao of Inner Mongolia were grouped into one cluster, and platycodin D content was higher in *P. grandiflorum* from these regions. The second category mainly included Shaanxi, Shandong, Inner Mongolia, Anhui, and Hebei. The third category is mainly Shaanxi and Henan. The remaining category is two samples from Anhui, their content is low, and they are all from e-commerce.

Platycodin D content was positively correlated with that of total platycodins (as platycodin D, platycodin D_3_, and platycoside E) (P < 0.05). The chemical structures of platycodin D, platycodin D_3_, and platycoside E are shown in Supplementary Fig. S4. The content of samples from the Chifeng area was simultaneously determined, and the results (Supplementary Table S4) similarly showed that platycodin D content was positively correlated with total platycodin content (P < 0.05). Therefore, we speculate that there is a potential association between platycodin D content and total platycodins content and that changes in other components may be determined indirectly by measuring platycodin D content to provide a basis for the quality of *P. grandiflorum*.

### Industrial structure and VCs

**Figure 2:**
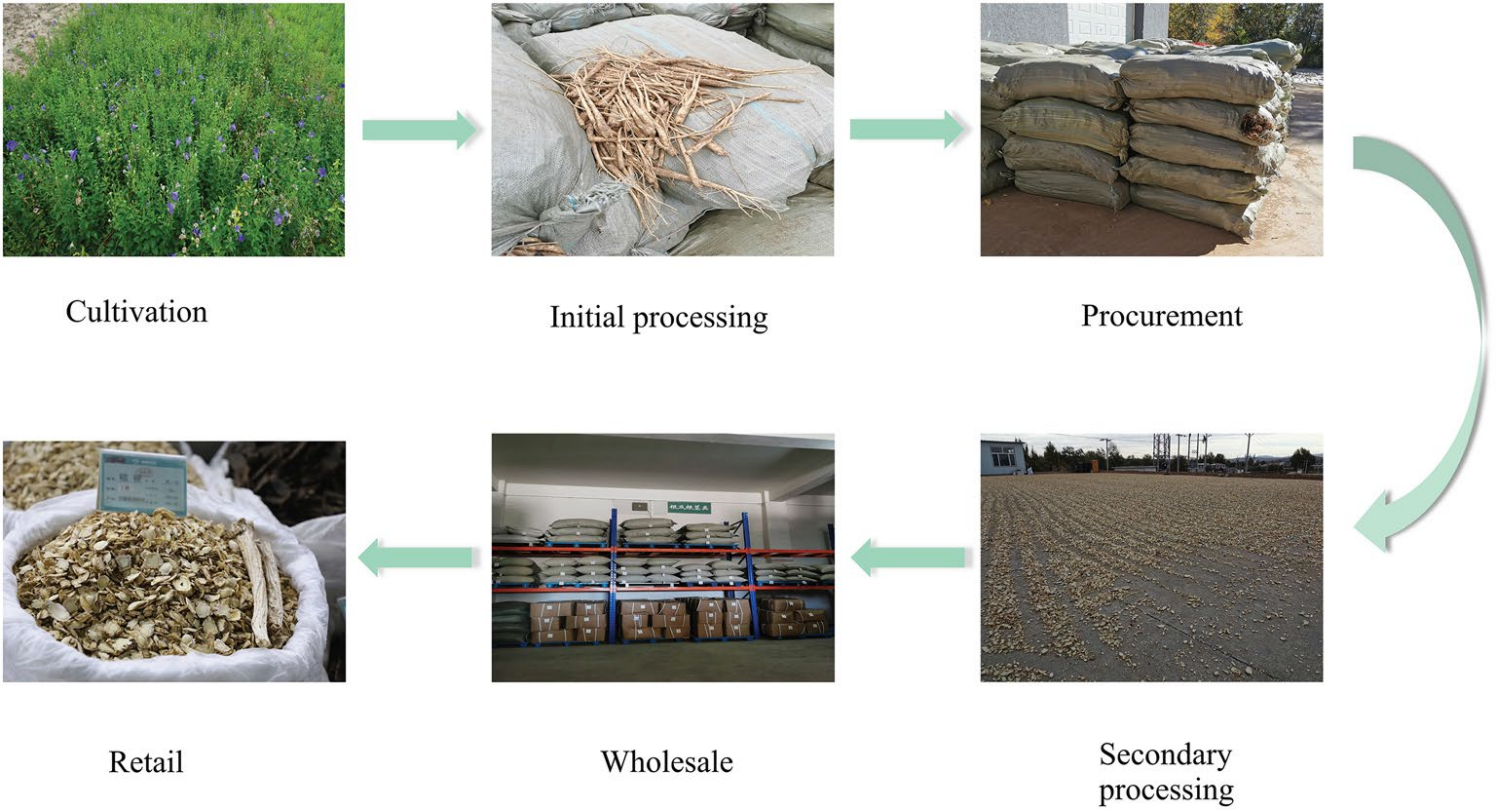
*P. grandiflorum* goes through six production stages in VC.

Most often, *P. grandiflorum* undergoes six processing stages before reaching consumers (Fig. 2): (1) Cultivation, including planting, irrigation, field management, pest control, and harvesting; (2) Preliminary processing or rough processing of fresh medicinal materials, including surface treatment of new medicinal materials to remove impurities; (3) Acquisition or purchase, i.e., the transaction of unprocessed *P. grandiflorum* produced in the second step; (4) Deep processing, including peeling, slicing, drying, and grading packaging; (5) Wholesale, mainly for food and medicinal materials market; (6) retail.

**Figure 3:**
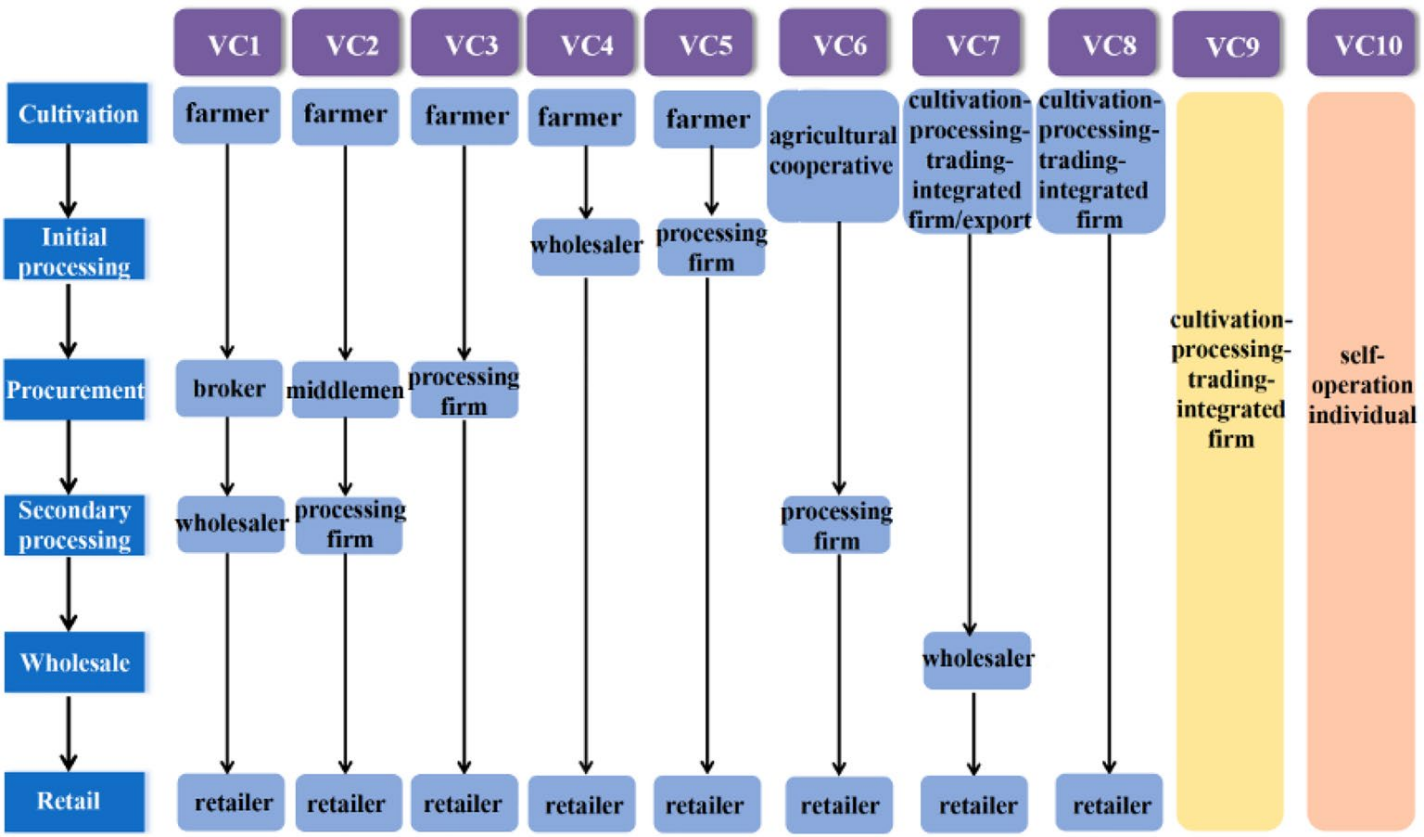
Primary VCs and stakeholders involved in *P. grandiflorum* produce (We obtained quality and risk information on *P. grandiflorum* under different VCs from farmers, middlemen, wholesalers, retailers, and firms in Fig. 3).

As the production and supply lines of *P. grandiflorum* exist in several forms, our survey revealed that stakeholders play different roles at each step, resulting in 10 VCs of different forms. The 10 main VCs identified were related to *P. grandiflorum,* and the roles of related stakeholders are shown (Fig. 3).

VCs 1–2 start with independent farmers with relatively small farms and are particularly common in places far from the markets for traditional Chinese medicines, such as Chifeng in Inner Mongolia. Due to the insufficient processing capacity of local companies, local consumption is also limited. Therefore, through the intermediary role of middlemen or brokers, fresh harvested medicinal materials or preliminarily processed *P. grandiflorum* are sold to pharmaceutical wholesaler processing companies such as Anguo or food processing companies such as Shandong and are further circulated throughout the country. *P. grandiflorum* is used as food and is sterilized, shredded, and processed into pickles. The processed pickles are packaged into small packages, sold in supermarkets or farmers' markets, or exported to Korea.

VCs 3–5 are different from VCs 1–2. These are the results of the business expansion of processing enterprises. Thanks to the convenience of the local trade center, numerous processing companies can purchase raw or preliminary *P. grandiflorum* directly from farmers. Processing enterprises can purchase directly from the local market and nearby production areas, thus increasing ease of use and flexibility. In addition, farmers close to traditional herbal medicine markets or processing companies can sell their products directly. This model is prevalent in traditional medicine markets, where local farmers can sell *P. grandiflorum* directly to wholesalers. Moreover, processing companies in the Chifeng producing area will also sign agreements to purchase *P. grandiflorum* from local farmers, which reduces the price increase of middlemen and increases the profits associated with cultivating *P. grandiflorum*.

VC 6 starts with agricultural cooperatives consisting of larger farmland. Participating farmers will receive production means processing facilities and production technology training from cooperatives. They receive encouragement and preferential policies from relevant government departments and easily benefit from financial and technical support from local governments.

VCs 7, 8, and 9 are controlled by companies with vertical integration of plant-processing trade, which involves vertical coordination within the industrial chain. These companies have a larger planting base and a high degree of mechanization. They are involved at all points of the VC, from production to processing to wholesale, thus, bridging the gap between farmers and trading companies or other production enterprises. Their products are sold to out-of-town retailers or consumers through chain stores or online stores. All stages are traceable, including the production chain, planting techniques, and quality inspection, as there are fewer intermediate links between wholesalers and retailers; thus, reducing costs and improving margins. In this model, companies can also work with local farmers to provide farmers with production targets as well as standards before the annual production process begins and determine the current purchase price.

VC 10 is a fully integrated VC in which the self-employed individuals might employ local farmers or carry out production and processing by themselves, where these individuals have relatively large farmlands. In this scenario, the products are often sold through their own physical and online stores.

We analyzed the quality of *P. grandiflorum* in different VCs (Table 1). The data on heavy metals and agricultural residues presented in Table 1 are from 48 samples of *P. grandiflorum* collected in the VC survey (Supplementary Table S6). The contents of total Pd, Cd, As, Hg, and Cu varied between 0 and 0.0961 mg/kg, 0 and 0.0287 mg/kg, 0.0803 and 0.2416 mg/kg, 0 and 0.0512 mg/kg, 4.4993 and 8.4028 mg/kg, with an average concentration of 0.0449, 0.0082, 0.1173, 0.0151, and 6.4539 mg/kg, respectively. The residues of BHC, DDT, and PCNB from the measured pesticide residues were below 0.0080, 0.0022, and 0.0012 mg/kg, below permissible limits. According to the 2020 edition of *Chinese Pharmacopoeia*, lead in medicinal materials and prepared drugs in pieces (plants) should not exceed 5 mg/kg, cadmium should not exceed l mg/kg, arsenic should not exceed 2 mg/kg, mercury should not exceed 0.2 mg/kg, copper should not exceed 20 mg/kg, and prohibited pesticides should not be detectable (Do not exceed the quantitative limit)^9^. All 48 samples were in accordance with the regulations of *Chinese Pharmacopoeia* on heavy metals and agricultural residues. The quality of *P. grandiflorum* with different VC values and the possibility of quality risk in the production process were analyzed by interviewing different stakeholders whether they had established production files, including seed sources, planting requirements, cultivator information, fertilization information, pesticide use information, harvest information, transportation information, storage, and sales information. In VCs 1–5, the quality of *P. grandiflorum* was unstable during transportation because it was sold directly by farmers without drying treatment. There is also a lack of effective quality supervision in VC 6. In VCs 7–10, an integrated industrial chain ensures quality and reduces waste.

**Table 1:** Quality of the *P. grandiflorum* for the different VCs and likelihood of risks being made to its quality during its production.

VC	Traceability	Certify	Control	Heavy metal	Pesticide residue	Likelihood of hazard occurring		
						Cultivation	Processing	Wholesale
1	Untraceable	Maybe	Medium	No risk	No risk	Improbable	Probable	Probable
2	Untraceable	No certify	Weak	No risk	No risk	Improbable	Very probable	Very probable
3	Untraceable	No certify	Weak	No risk	No risk	Improbable	Very probable	Very probable
4	Untraceable	Maybe	Medium	No risk	No risk	Improbable	Probable	Probable
5	Untraceable	Maybe	Medium	No risk	No risk	Improbable	Probable	Very probable
6	Untraceable	No certify	Weak	No risk	No risk	Improbable	Probable	Probable
7	Untraceable	No certify	Weak	No risk	No risk	Improbable	Probable	Probable
8	Maybe	Maybe	Strong	No risk	No risk	Improbable	Improbable	Improbable
9	Traceable	Maybe	Strong	No risk	No risk	Improbable	Improbable	Improbable
10	Traceable	Maybe	Strong	No risk	No risk	Improbable	Improbable	Probable

### Food chain analysis

#### Anti-cancer activity of *P. grandiflorum* extract using different links

**Figure 4:**
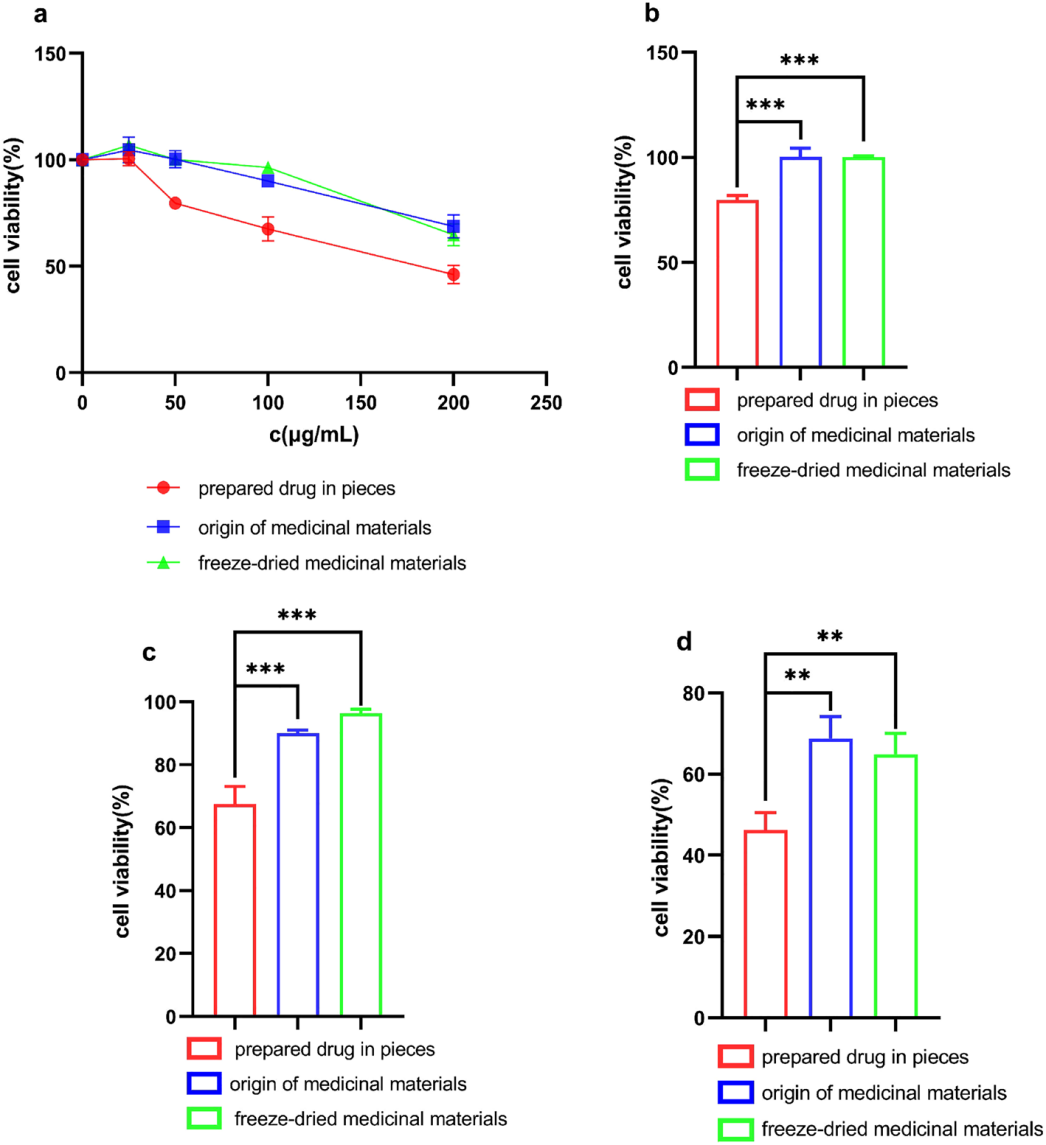
The effect of *P. grandiflorum* extract on A549 cell viability. ((**a**) The effect of *P. grandiflorum* extract on A549 cell viability at different concentrations; (**b**) The effect of *P. grandiflorum* extract on A549 cell viability at 50 μg/mL concentrations; (**c**) The effect of *P. grandiflorum* extract on A549 cell viability at 100 μg/mL concentrations; (**d**) The effect of *P. grandiflorum* extract on A549 cell viability at 200 μg/mL concentrations) **P < 0.01; ***P < 0.001.

The results of CCK-8 showed that the inhibitory effect of different crude extracts on the proliferation of A549 cells increased dose-dependent (Fig. 4a). These results indicated that *P. grandiflorum* extract had a strong inhibitory effect on A549 cell proliferation in vitro, and we preliminarily determined that *P. grandiflorum* extract had certain anti-lung cancer activity. In addition, by comparing the inhibitory effect of different extracts on A549 cells, we discovered that *P. grandiflorum* prepared drug in pieces had a more significant inhibitory effect on A549 cells. Compared with original medicinal materials and freeze-dried medicinal materials, the concentration of 50 μg/mL, 100 μg/mL, and 200 μg/mL prepared drug in pieces treatment of *P. grandiflorum* extract (Fig. 4b–d) significantly inhibited the viability of A549 cell. (**P < 0.01; ***P < 0.001).

#### Anti-inflammatory activity of *P. grandiflorum* extract using different links

**Figure 5:**
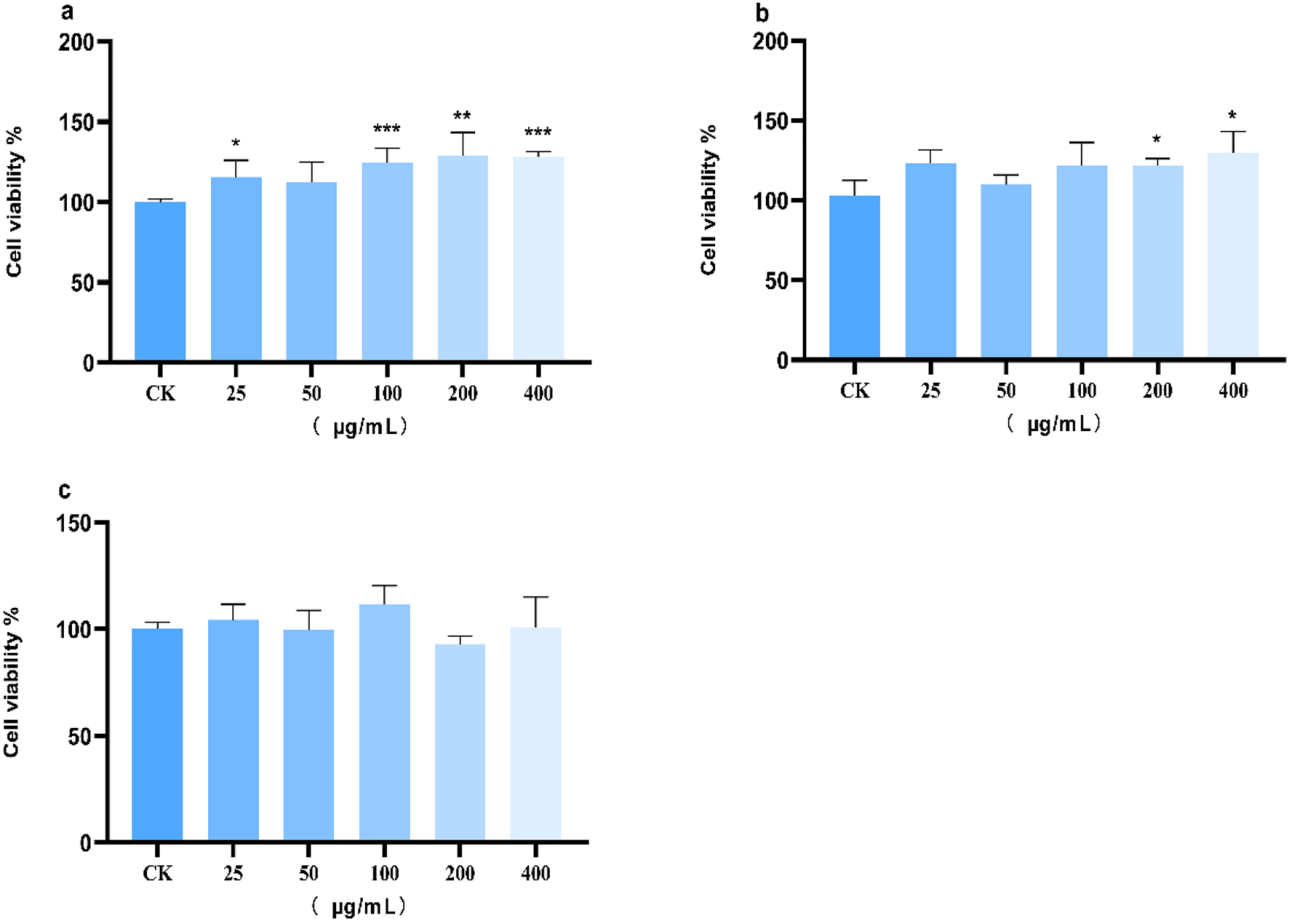
The effect of *P. grandiflorum* extract on RAW264.7 cell viability. ((**a**) Effects of different concentrations of original medicinal materials extracts on the activity of RAW264.7 cells; (**b**) Effects of different concentrations of prepared drug in pieces extracts on the activity of RAW264.7 cells; (**c**) Effects of different concentrations of freeze-dried medicinal materials extracts on the activity of RAW264.7 cells; CK: control check) *P < 0.05; **P < 0.01; ***P < 0.001.

Compared to the control group, the original medicinal materials treatment of *P. grandiflorum* at concentrations of 25, 100, 200, and 400 μg/mL they significantly affected RAW264.7 cell proliferation (P < 0.05) (Fig. 5a). The prepared drug in pieces of *P. grandiflorum* (200 and 400 μg/mL) led to significant differences in RAW264.7 cell proliferation (P < 0.05) (Fig. 5b). For freeze-dried medicinal materials, there was no significant difference in RAW264.7 cell proliferation at 25, 50, 100, 200, and 400 μg/mL (P > 0.05) (Fig. 5c). Overall, the different *P. grandiflorum* extracts exerted no toxic effects on RAW264.7 cells within the experimental concentration range. Thus, subsequent experiments were conducted using 25, 50, 100, 200, and 400 μg/mL concentrations.

**Figure 6:**
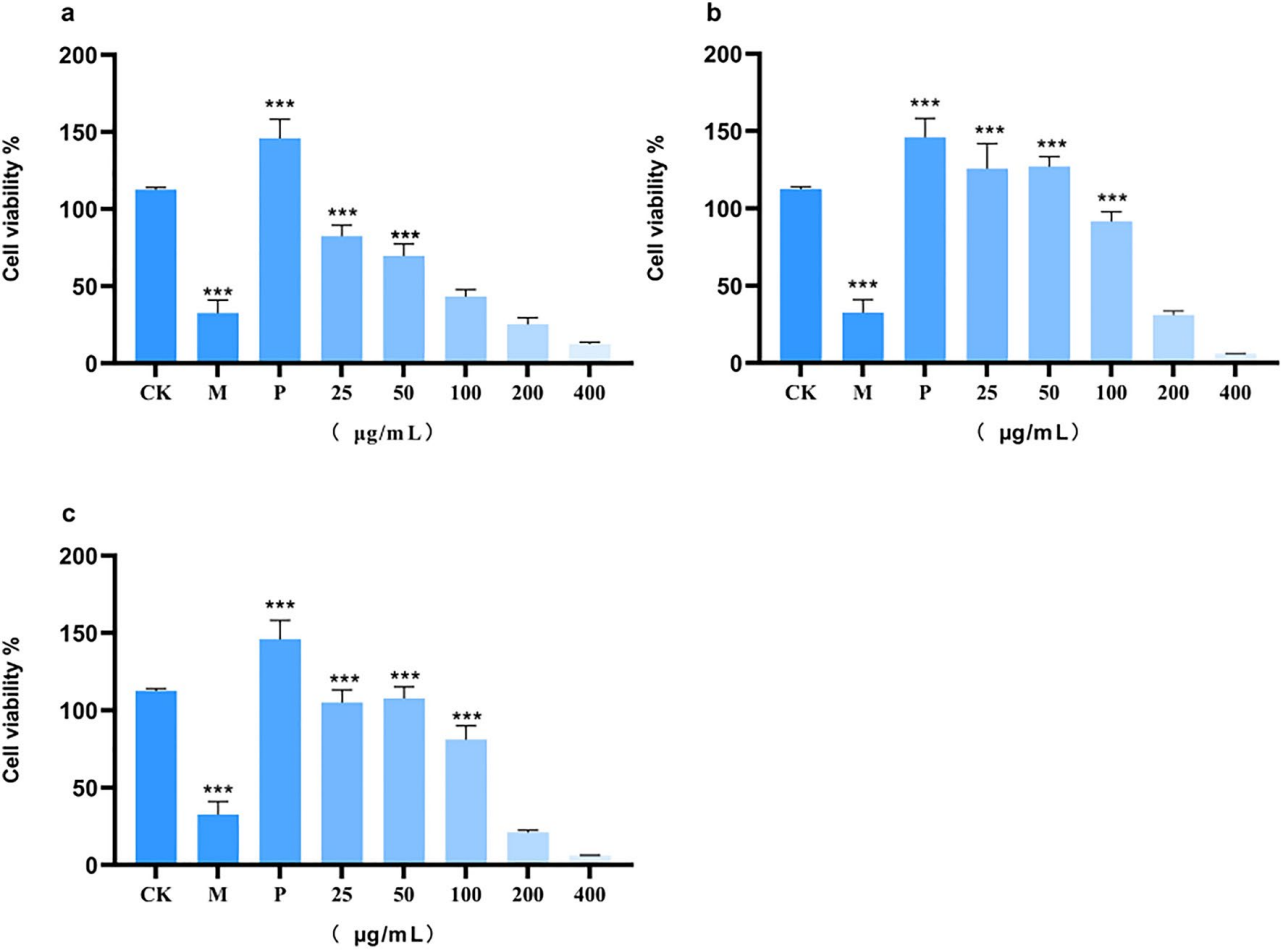
Protective effects of different treatments of *P. grandiflorum* on lipopolysaccharide induced RAW264.7 cell inflammation ((**a**) Protective effects of different concentrations of original medicinal materials on lipopolysaccharide induced RAW264.7 cell inflammation; (**b**) Protective effects of different concentrations of prepared drug in pieces on lipopolysaccharide induced RAW264.7 cell inflammation; (**c**) Protective effects of different concentrations of freeze-dried medicinal materials on lipopolysaccharide induced RAW264.7 cell inflammation; CK: control check; M: model group; P: positive control group) ***P < 0.001.

The survival rate of the LPS model group was significantly reduced (P < 0.001) (Fig. 6). The cell survival rate increased after treatment with the positive control drug indomethacin, and the difference was significant compared with the untreated group (P < 0.001). Compared with the LPS model group, original medicinal materials treatment of *P. grandiflorum* extract (Fig. 6a) at 25 and 50 μg/mL, prepared drug in pieces treatment of *P. grandiflorum* extract (Fig. 6b) at 25, 50, and 100 μg/mL, and freeze-dried medicinal materials treatment of *P. grandiflorum* extract (Fig. 6c) at 25, 50, and 100 μg/mL significantly ameliorated the inflammatory effect of LPS on RAW264.7 cell (P < 0.001); thus, this indicates that different treatments with *P. grandiflorum* extract can protect RAW264.7 cells against LPS-induced inflammation. Among them, treatment with *P. grandiflorum* extract from the prepared drug in pieces showed the strongest activity.

#### Transfer efficiency of active components in the food chain of *P. grandiflorum* for each nutrient level

**Figure 7:**
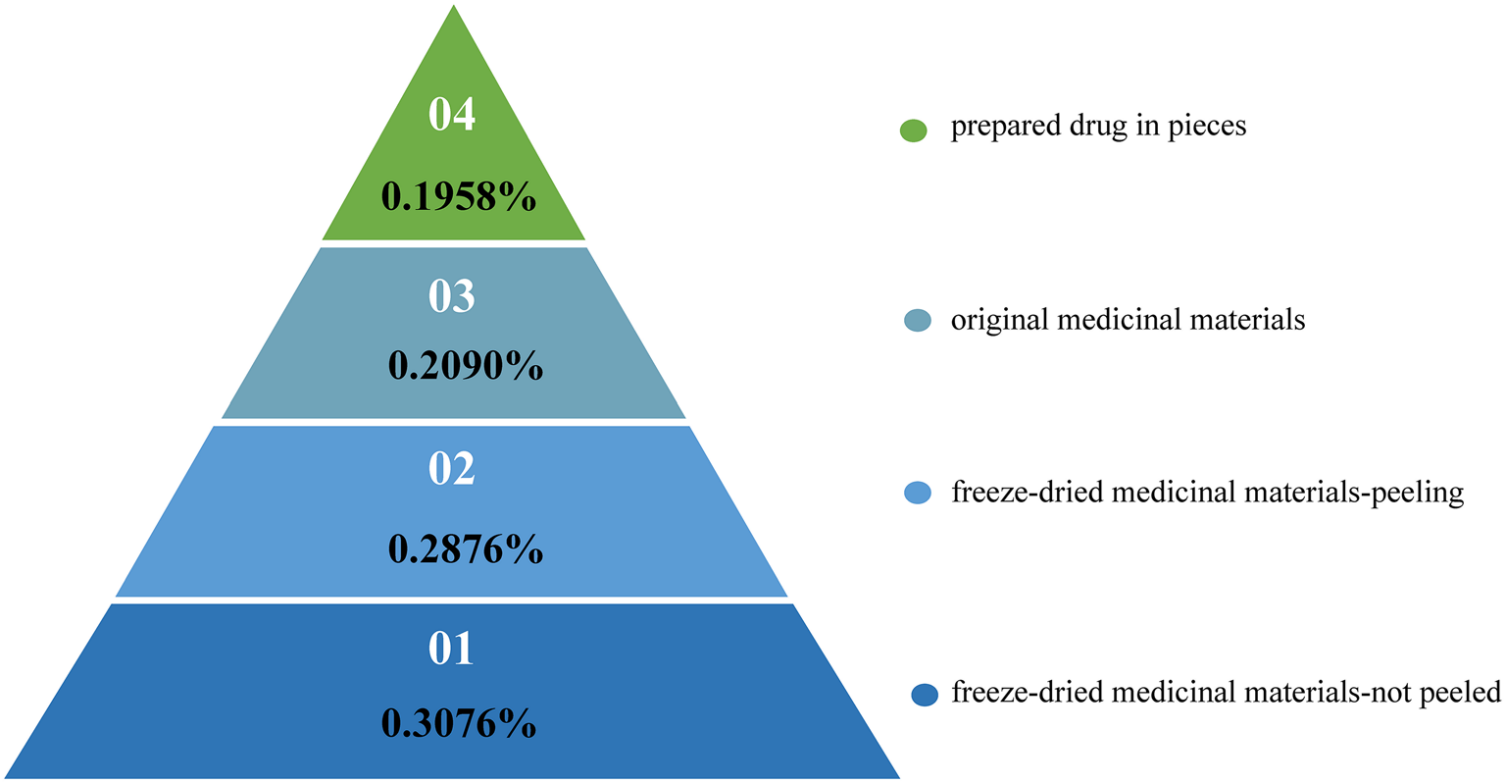
The results of the active components transfer efficiency of four main nutrient levels associated with *P. grandiflorum*.

Our survey revealed that different nutrient levels play different roles at each link, suggesting that *P. grandiflorum* in these four nutritional levels is likely to be sold directly to consumers. The results of the active components (platycodin D %) of the four main nutrient levels associated with *P. grandiflorum* are summarized in Supplementary Table S4. The results of the active components transfer  efficiency of four main nutrient levels associated with *P. grandiflorum* are shown (Fig. 7). Transfer efficiency of active components in the four nutritional levels from more to less were freeze-dried medicinal materials, original medicinal materials, and prepared drug in pieces. This is consistent with the theory that energy decreases as the food chain flows.

## Discussion

This study determined the platycodin D content in *P. grandiflorum* samples collected during the VC survey. The content of platycodin D in 48 samples ranged from 0.12% to 0.38%, with an average of 0.23%. The platycodin D content in the samples was concentrated at 0.20%–0.30%, among which the high content of platycodin D was mainly concentrated in Chongqing, Sichuan, and Shanxi Province as well as Tongliao City in Inner Mongolia. The two batches with low content of platycodin D were mainly distributed in the Anhui Province. However, we discovered that both samples came from e-commerce, which led to the uncertainty of sample quality. Platycodin is the main indicator component in *P. grandiflorum,* according to the 2020 edition of the *Chinese Pharmacopoeia,* and the content of platycodin D is not less than 0.10%^9^. The content of platycodin D in *P. grandiflorum* samples from all regions complies with pharmacopoeia regulations, explaining why all VCs are circulating in the market. Other studies also showed that the quality of most *P. grandiflorum* and decoction pieces circulating in the market was reasonable; for example, the content of platycodin D in Anhui, Shandong, Inner Mongolia, Gansu, Yunnan, Sichuan, and other places was higher than 0.10%. The average content of platycodin D in East China was more than 0.20%^30^. However, the content of a single chemical component cannot guarantee the sustainability of the *P. grandiflorum* industrial chain, and further analysis is needed to analyze the relationship between *P. grandiflorum* quality and different stakeholders. Since multiple indexes can comprehensively evaluate the quality and interaction between components, we discovered that platycodin D is the main active ingredient, while platycodin D, platycoside E, and platycodin D_3_ have similar structures and diverse pharmacological activities; thus, we performed a literature analysis. Based on our literature analysis, we concluded that platycodin D was positively correlated with total platycodins. We also conducted experiments to verify the positive correlation between platycodin D and total platycodins. However, the change in the content of various chemical compositions has not been confirmed, and the literature and content determination alone are insufficient to verify this relationship. There were also studies on the relationship between platycodin D and total platycodins content in *P. grandiflorum* for different years of cultivation, and the results showed no correlation^31^. The content changes of various saponins in *P. grandiflorum* are needed to evaluate the quality of *P. grandiflorum* more comprehensively. In the future, we will further study the correlation between the chemical components in medicinal materials and establish a more comprehensive quality control method, including the quality mark, ash content, and processing method of the water extract.

We have traceability, certification, quality control, agricultural residues, and heavy metals as indicators to analyze the quality risk in the production process of *P. grandiflorum*. The detection results of heavy metals and agricultural residues in *P. grandiflorum* samples were consistent with the 2020 edition of *Chinese Pharmacopoeia* standards for Hong Kong, Japan, Taiwan, the UK, and the USA. VC 1-5 is a chain that starts with the planting of independent farmers. Farmers are not restricted in planting. They plant according to their own planting experience and have no planting records, so the traceability of the entire VC of *P. grandiflorum* cannot be realized. In addition, the quality evaluation of *P. grandiflorum* by farmers is qualitative, based on the parameters such as appearance, size, and presence of mold, among others, and the quality of *P. grandiflorum* cannot be well controlled. Although VC 6 started from an agricultural cooperative composed of larger farmland, which reduced the ability of farmers to bear risks alone, the quality control of *P. grandiflorum* within the cooperative is not strict, and there is no uniform standard to implement. The addition of wholesale in VC 7 also increases the uncontrollable factors of the quality of *P. grandiflorum*, and we cannot ensure that the storage of *P. grandiflorum* by wholesale meets the regulations. VC 8-10, the firm covers the whole link from planting to sales, which is convenient for vertical management within the enterprise, and requires farmers to carry out standardized cultivation of *P. grandiflorum* in accordance with relevant standards. For example, "The cultivation technical specification of Radix Platycodonis in eastern Inner Mongolia" (DB 15/T 1137—2017) regulates the environment of the producing area, land selection, land preparation, sowing and raising seedlings, field management, disease and pest control, and harvesting among others. In addition, the firm also carries out chemical identification, quantitative analysis, and quantitative quality control of pesticide and heavy metal residues on *P. grandiflorum* in accordance with pharmacopoeia and relevant standards. Therefore, the traceability of VC8- VC10 is high, the risk is low, and it is worth further promotion.

Currently, *P. grandiflorum* is used mainly in two markets for medicinal materials and food. The main sales channels include medicinal materials markets, drugstores, hospital pharmacies, food markets, and online stores. In VCs 2 and 7, *P. grandiflorum* is sold primarily as food. From the food VC perspective, appearance, character and taste, growth thickness, white skin, and the fresh meat of *P. grandiflorum* are considered good quality indicators. Furthermore, in the food chain, due to the high requirements on appearance and traits, stout and straightforward products are selected as food supply through simple processing and classification; thus, the quality and price of these *P. grandiflorum* products are often higher than those of traditional medicine market products. Our investigation revealed that due to natural climate and other favorable factors, *P. grandiflorum* in Chifeng, Inner Mongolia, has certain advantages in appearance and taste, which makes the price of *P. grandiflorum* higher in the Chifeng area. Studies on the VC of *Cistanche deserticola*^19^, *Lycium barbarum*^20^, *Astragalus membranaceus* var. *mongholicus*^22^, and *Glehnia littoralis*^32^ also show similar results. The VC with vertical integration of plant-processing trade shows advantages in all VCs. It is worthy of promotion and sustainable development from the perspective of farmers' income and the quality of *P. grandiflorum*.

In the VC of *P. grandiflorum*, different links have different effects on quality. In the planting process, due to the lack of unified planting technology and field management standards in different producing areas, the product quality of these VCs is seriously affected, which also limits the consistency of *P. grandiflorum* harvest. In terms of safety, most products in different links of the *P. grandiflorum* VC are safe because the actual planting process is subjected to the quality standards imposed by the Provisions of The *Chinese Pharmacopoeia*^9^ and the implementation of the zero-growth policy of pesticides in recent years. In the link of *P. grandiflorum* trading, fresh goods are usually 2 yuan/g, unpeeled dry sub-goods are 8 yuan/kg, and dry peeling is 15 yuan/kg. Due to the labor associated with drying and processing, farmers often sell fresh *P. grandiflorum,* which is associated with a low expected income. Small farmers often look for ways to reduce the production cost and increase their margins, which affects the quality of the medicinal materials, eventually leading to their products entering the low-end market and reducing their profits. In addition, in VCs 7–9, these enterprises pay attention to deep processing and use various processing methods to develop the product's added value, which allows them to cater to a larger market and achieve higher profits. For pharmaceutical enterprises but not independent farmers, the quality of traditional Chinese medicine can be traced, and their marketing is more diversified, which can also lead to greater profits. In addition, the brand stability and increased drug prices from foreign markets incentivize enterprises to spend more time and energy to trace the quality of *P. grandiflorum* and ensure that their products meet the inspection standards required by the foreign markets, which brings high-profit returns to stakeholders. Through e-commerce platforms, VC 10 medicinal materials can be sold, greatly reducing the costs associated with physical stores. However, there are several gaps in the supervision process, and some non-standard and uncontrollable links may appear. Therefore, in general, the behaviors and interests of stakeholders, as well as product quality and target market, are closely related.

From the food chain perspective, the quality of *P. grandiflorum* was evaluated by calculating the transfer efficiency of effective components of *P. grandiflorum* of different nutrient levels. Transfer efficiency of effective components of *P. grandiflorum* in the order of more to less is freeze-dried medicinal materials, original medicinal materials, and prepared drug in pieces. Nevertheless, in our experiments, the anti-inflammatory activity of prepared drugs in pieces was the strongest. Similarly, *P. grandiflorum* prepared drug in pieces showed the most potent activity inhibiting cancer cell activity and proliferation. Regarding the nutritional levels of the prepared drug in pieces, the transfer efficiency of platycodin D was the lowest During the wetting process, *P. grandiflorum* may lose a certain amount of water-soluble components, and platycodin D is also a part of it, leading to the lowest platycodin D content in decoction pieces^30^. In addition, the contents of Deapi-platycoside E, platycoside E, platycodin D_3,_ and Desapioplatycodin D in the original medicinal materials were also higher than those in decoction pieces^30^. The content of platycoside E in the prepared drug in pieces determined in this paper was also lower than the original medicinal materials. The researchers carried out a study on the processing technology of *P. grandiflorum*. The content of platycodin D in the original medicinal materials was 0.48%, and platycodin D in the traditionally processed slices was reduced to 0.25%, but platycodin D in the processed slices, while fresh, could reach 0.44%^33^. The process of fresh processing eliminates the steps of processing *P. grandiflorum* from fresh products into medicinal materials, softening medicinal materials, and reducing the loss of active ingredients. Therefore, fresh processing has certain feasibility and superiority^33^. The alcohol extract of *P. grandiflorum* contains saponins, flavonoids, polysaccharides, polyphenol, and other components^34^. A neutral heteropolysaccharide isolated from *P. grandiflorum* significantly stimulated the proliferation of RAW264.7 cells in the range of 100 μg/mL to 800 μg/mL (P < 0.05), enhanced its phagocytic ability and increased in a dose-dependent manner^35^. Ethanol extract and fermented extract of *P. grandiflorum*^17^ significantly reduced NO concentration in LPS-induced RAW 264.7 cells in a dose-dependent manner (P < 0.05)^36^. In addition, the methanol extract of *P. grandiflorum* contains phenolic components, which play an anti-inflammatory role by inhibiting the production of proinflammatory cytokines IL-6, IL-12p40, and TNF-α in mouse RAW264.7 macrophages stimulated by lipopolysaccharide^37^. Platycodin D can induce autophagy in A549 cells by inhibiting PI3K/Akt/mTOR signaling pathway, activating JNK and p38 MAPK signaling pathways^38^. In addition to platycodin D, other chemical components also have anti-tumor effects. The levels of LC3-II increased over time and in a dose-dependent manner in A549 cells treated with PGB (a platycoside-enriched butanol fraction of PG), suggesting that PGB-induced autophagy inhibits cell viability^39^. Platycodon A and Platycodon B were tested in A549 cancer cell lines and showed remarkable cytotoxic activities against A549 cancer cell lines with IC50 values ranging from 4.9 to 9.4 µM^40^. The water extract of *P. grandiflorum* also inhibited the growth and induced apoptosis of A549 cells in a dose-dependent manner^41^. Both platycodin D_3_ and platycodin D have anti-inflammatory, expectorant, antitussive, and anti-tumor activities^42^. These studies also suggest that not only platycodin D, but also polyphenols, polysaccharides and other saponins play an anti-inflammatory and anti-cancer role in *P. grandiflorum* extract. This also explains why the delivery efficiency of platycodin D content in *P. grandiflorum* is different from the results of anti-cancer and anti-inflammatory activities of *P. grandiflorum* extract. One of the characteristics of traditional Chinese medicine is integrity, and the main effective components of *P. grandiflorum* are saponins, so platycodin D_3_, platycoside E, and other components should also play a certain role in the efficacy of *P. grandiflorum*. However, there was no study on the content changes of saponins, polysaccharides, and polyphenols in *P. grandiflorum* processed into pieces. The activity of prepared drugs in pieces is better than the original medicinal materials, which may be closely related to the extraction and processing methods. Therefore, research on the food chain of *P. grandiflorum* should consider the content of multiple chemical components as indicators and clarify the ratio relationship of various active ingredients by determining the relationship between the content of active ingredients, processing methods, and pharmacological activities to provide more comprehensive indicators for the quality control of *P. grandiflorum* along the VC. To better explain the food chain of *P. grandiflorum*—the active components and activities in *P. grandiflorum* change along the chain.

## Conclusion

This study focused on VCs and the quality of *P. grandiflorum* and showed that the stakeholders in the VC have a certain impact on the quality and price of crude drugs. By combining cultivation and processing, a more efficient, standardized, and stable supply chain can be achieved, and the origin of substandard medicinal materials can also be traced back. Therefore, it is necessary to develop a cooperation mode between enterprises and farmers that relies on a strict and complete planting and purchasing system to ensure the marketability and traceability of the collected medicinal materials and their source. In other words, ensuring the quality and safety of *P. grandiflorum* is the cornerstone of developing its market, which is conducive to the further advancement of medicine food homology products.

Herein, we showed that the quality of *P. grandiflorum* in the VC is volatile and controllable. By coordinating the relationship within the venture capital company, further integration of resources is conducive to ensuring the quality of *P. grandiflorum* and increasing stakeholders' income. Lastly, we analyzed the processing links of *P. grandiflorum* from the perspective of the food chain and compared the active ingredients and pharmacological activities of different links related to the VC of *P. grandiflorum*. We found that the processing of *P. grandiflorum* prepared drug in pieces had a good preservation effect and involved various chemical components; moreover, the content of a single chemical component could not represent the efficacy of *P. grandiflorum*, and more in-depth studies are required.

### Supplementary Information

Supplementary Information.

## Data Availability

All data generated or analysed during this study are included in this published article [and its supplementary information files].
