# Supplementary material for: Quality control of Platycodon grandiflorum (Jacq.) A. DC. based on value chains and food chain analysis
Source: Sci Rep. 2023 Aug 28;13:14048. doi: 10.1038/s41598-023-41013-8 (PMC10462715; doi:10.1038/s41598-023-41013-8)
Supplement: Supplementary file 1 — Supplementary Information. [file 41598_2023_41013_MOESM1_ESM.pdf]

Quality control of *Platycodon grandiflorum* (Jacq) A. DC based on value chains and food chain analysis

Linlin Jiang[1,2] †, Hui Niu[1,2,3]†, Yuan Chen[1,2], Xing Li[1,2,3] , Yulian Zhao[1,2,3], Chunhong Zhang[3*], Minhui Li[1,2,3,4*]

[1] Inner Mongolia Hospital of Traditional Chinese Medicine, Hohhot 010020, China; jianglinlin27@163.com (L.J.); jy13190517573@163.com (H.N.); chenyuan9711@163.com (Y.C.); lx15704967759@163.com (X.L.); z18330990760@163.com (Y.Z.)

[2] Inner Mongolia Traditional Chinese & Mongolian Medical Research Institute, Hohhot 010010, China

[3] Department of Pharmacy, Baotou Medical College, Baotou 014040, China

[4] Inner Mongolia Key Laboratory of Characteristic Geoherbs Resources Protection and Utilization, Baotou 014040, China

* Correspondence: prof_liminhui@yeah.net (M.L.); Tel.: 86-471-416 0716 (M.L.); zchlhh@126.com (C.Z.); Tel.: 86-472-716 7795

† These authors contributed equally to this work and share first authorship

Supplementary Information

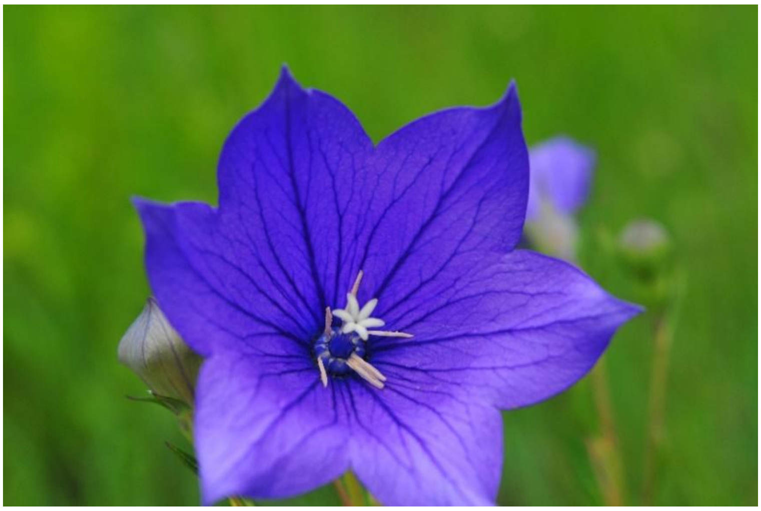

Figure S1. Flowering *Platycodon grandifloras* (The photo was taken by Minhui Li, the corresponding author of this article)

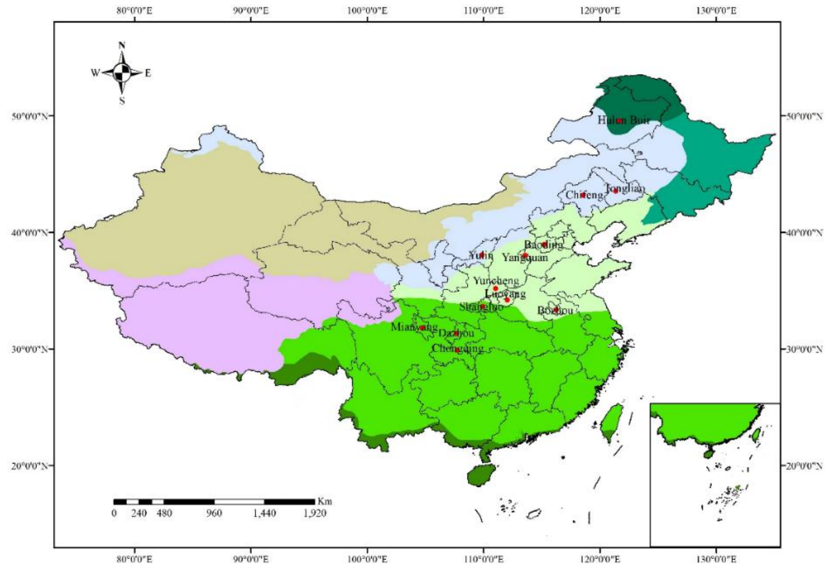

Figure S2. The collection place of *P. grandiflorum* sample (This map was created by the author of this article, Yuan Chen, using version 10.8 of ArcGIS. http://xdc.at/map/wmts)

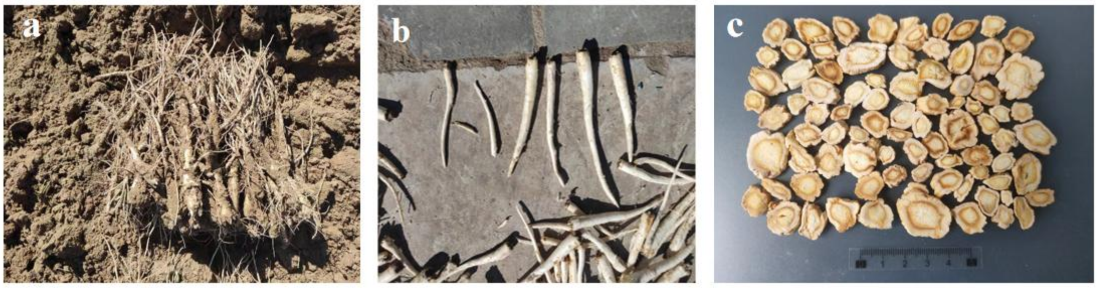

Figure S3. *P. grandiflorum* for value chain analysis
(a: fresh raw *P. grandiflorum*; b: original medicinal materials; c: prepared drug in pieces)

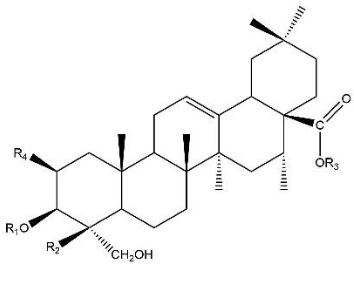

	R_1	R_2	R_3	R_4
1 Platycodin D	¹Glc-	CH_2OH	¹Ara²-¹Rha⁴-¹Xyl³-¹Api	OH
2 Platycodin D_3	¹Glc⁶-¹Glc-	CH_2OH	¹Ara²-¹Rha⁴-¹Xyl³-¹Api	OH
3 Platycoside E	¹Glc⁶-¹Glc⁶-¹Glc-	CH_2OH	¹Ara²-¹Rha⁴-¹Xyl³-¹Api	OH

Figure S4. Chemical structures of platycodin D, platycodin D_3, and platycoside E

Table S1 The application of *P. grandiflorum* in food, cosmetics, medicine and food health products

category	product
food	*P. grandiflorum* sauerkraut [1]; *P. grandiflorum* rice vinegar [2]; *P. grandiflorum* wine [3]; *P. grandiflorum* fermented drink [4]; *P. grandiflorum* fruit [5]; *P. grandiflorum* cookie [6]
cosmetics	whitening essence; whitening essence milk [7]
food health products	*P. grandiflorum* complex health powder [8]; *P. grandiflorum* phlegm health lozenges [9]; *P. grandiflorum* polysaccharide oral liquid [10]
Medicine	*P. grandiflorum* decoction (Synopsis of Golden Chamber); *P. grandiflorum* tablets (Jilin Provincial Drug Standard, 1986), Chuankening (Heilongjiang Provincial Drug Standard, 1986).

References

[1] Li, F. Y. & Zhou Q. C. Processing technology of Platycodon root health pichle of low salt. *China Condiment*. **33**, 59-60, 65 (2008).

[2] Shi, P. N., et al. Brewing and quality analysis of flavored Radix platycodonis rice vinegar. *China Condiment*. **46**, 95-100, 116 (2021).

[3] Li, D. Q. & Wang, X. J. Development of Campanulaceae and orange peel wine. *Liquor Making Science & Technology*. 87-89 (2011).

[4] Yu, J., Zhang, X. H. & Cheng, P. *Platycodon grandiflorum* fermentation healthcare beverage processing method: CN106360224A. 2019-05-03.

[5] Zhao, L. Q., Wang J. J. & Yang, X. J. Characteristics of dried fruit development[J]. *Inner Mongolia Agricultural Science and Technology*. 32-33 (2002).

[6] Zhang, X. L. et al. Method for preparing *Platycodin grandiflora* as cookies: CN110754503A. 2020-02-07

[7] Zhao, S. T., Tang, M. M., Zhen, X. X. & Hou, W. History of *platycodon grandiflorum* processing, product development and modern research progress. *Specialty Res*. 507-513 (2023)

[8] Zhu, N. N. Manufacture method for *Platycodon grandiflorum* composite healthcare powder: CN105361145A. 2019-04-30.

[9] Tai, J. et al. Study on the preparation technology of *Platycodon grandiflorum* health buccal tablets. *Shandong Chemical Industry*. **44**, 46-48 (2015).

[10] Liu, C. Development of Platycodon polysaccharide oral liquid based on orthogonal test. Contemporary Chemical Industry. **45**, 2097-2099. (2016).

Table S2 Questionnaire for *P. grandiflorum* used in fieldwork

respondent			
Age	Gender	Education level	Education level

P. grandiflorum acreage		Mu	
Cultivation method			
field management			
growing year			
harvesting methods			
Yield per Mu		Kg	
Origin of primary processing method			
Storage mode			
grading standards			
Character data measurement			
Application of *P. grandiflorum*		repertory	Kg
Way of selling	e-commerce; retail; order selling	Quality grade	green; non-pollution; organic
Product type	crude drug; decoction pieces	Number of workers	
Cost		Payroll	
Monthly turnover		Monthly income	
What makes good quality? What do you think is the most important technology for getting a high yield and good quality? Which is the most important?			
What does good quality *P. grandiflorum* look like? What is the relationship between quality and price?			
What do consumers care about?			
Do you know the price of other *P. grandiflorum* products? As well as the price of *P. grandiflorum* in other province? How? Can you use (do you have) smart phone or computer to visit the web?			
Are you satisfied with the present production model and why? What would you like to change to improve the situation? What kind of *P. grandiflorum* products will be more popular in the future			

What are the factors that affect the earnings of *P. grandiflorum*?

Table S3 The sampling information of *P. grandiflorum*

No.	Sample	Producing area	Processing	Collection time
1	Inner Mongolia-1	Inner Mongolia	Not peeled	2020.10.
2	Inner Mongolia-2	Inner Mongolia	Not peeled	2020.10.
3	Inner Mongolia-3	Inner Mongolia	Not peeled	2022.1.
4	Inner Mongolia-4	Inner Mongolia	Not peeled	2020.10.
5	Inner Mongolia-5	Inner Mongolia	Not peeled	2020.10.
6	Inner Mongolia-6	Inner Mongolia	Peeling	2021.10.
7	Inner Mongolia-7	Inner Mongolia	Peeling	2021.3.
8	Inner Mongolia-8	Inner Mongolia	Peeling	2021.3.
9	Inner Mongolia-9	Inner Mongolia	Not peeled	2021.4.
10	Inner Mongolia-10	Inner Mongolia	Not peeled	2021.4.
11	Inner Mongolia-11	Inner Mongolia	Peeling	2021.3.
12	Inner Mongolia-12	Inner Mongolia	Peeling	2022.1.
13	Inner Mongolia-13	Inner Mongolia	Peeling	2022.1.
14	Inner Mongolia-14	Inner Mongolia	Peeling	2022.1.
15	Anhui-1	Anhui	Peeling	2021.5.
16	Anhui-2	Anhui	Peeling	2021.5.
17	Anhui-3	Anhui	Peeling	2021.11.
18	Anhui-4	Anhui	Peeling	2021.11.
19	Anhui-5	Anhui	Peeling	2021.3.
20	Anhui-6	Anhui	Peeling	2021.3.
21	Anhui-7	Anhui	Peeling	2021.3.
22	Hebei-1	Hebei	Peeling	2020.11.
23	Hebei-2	Hebei	Peeling	2020.11.
24	Hebei-3	Hebei	Peeling	2022.1.
25	Hebei-4	Hebei	Peeling	2022.1.
26	Shaanxi-1	Shaanxi	Peeling	2021.4.
27	Shaanxi-2	Shaanxi	Peeling	2021.4.
28	Shaanxi-3	Shaanxi	Peeling	2021.4.
29	Shaanxi-4	Shaanxi	Peeling	2021.4.
30	Shaanxi-5	Shaanxi	Peeling	2021.4.
31	Shaanxi-6	Shaanxi	Peeling	2021.4.
32	Shaanxi-7	Shaanxi	Peeling	2021.3.
33	Shanxi-1	Shanxi	Not peeled	2020.12.
34	Shanxi-2	Shanxi	Peeling	2021.3.
35	Shanxi-3	Shanxi	Peeling	2021.11.
36	Sichuan-1	Sichuan	Peeling	2021.6.
37	Sichuan-2	Sichuan	Peeling	2021.6.
38	Sichuan-3	Sichuan	Peeling	2021.5.
39	Sichuan-4	Sichuan	Peeling	2021.5.
40	Chongqing-1	Chongqing	Peeling	2021.5.
41	Chongqing-2	Chongqing	Peeling	2021.5.
42	Chongqing-3	Chongqing	Peeling	2021.5.
43	Shandong-1	Shandong	Peeling	2021.5.
44	Shandong-2	Shandong	Peeling	2021.5.
45	Shandong-3	Shandong	Peeling	2021.5.
46	Shandong-4	Shandong	Peeling	2021.3.
47	Henan-1	Henan	Peeling	2021.3.
48	Henan-2	Henan	Peeling	2021.3.

Table S4 The platycodin E, platycodin D3, and platycodin D content (%) in *P. grandiflorum*

samples in Chifeng

Sample	platycodin E content (%)	platycodin D3 content (%)	platycodin D content (%)	Average platycodin D content (%)
freeze-dried medicinal materials-not peeled-1	0.3365	0.1039	0.1036	
freeze-dried medicinal materials-not peeled-2	0.3559	0.1001	0.2129	0.1656
freeze-dried medicinal materials-not peeled-3	0.3355	0.1001	0.1804	
freeze-dried medicinal materials-peeling-1	0.3191	0.1089	0.0591	
freeze-dried medicinal materials-peeling-2	0.3506	0.1007	0.2581	0.1548
freeze-dried medicinal materials-peeling-3	0.3726	0.1203	0.1472	
original medicinal materials-1	0.2875	0.1259	0.0642	
original medicinal materials-2	0.2732	0.1243	0.1790	0.1125
original medicinal materials-3	0.2586	0.1103	0.0944	
prepared drug in pieces-1	0.2882	0.1509	0.0596	
prepared drug in pieces-2	0.2988	0.1577	0.1322	0.1054
prepared drug in pieces-3	0.3057	0.1540	0.1244	

Table S5 The content of platycodin D in *P. grandiflorum* samples

No.	Content (%)	No.	Content (%)	No.	Content (%)
1	0.2507	17	0.2102	33	0.3414
2	0.2208	18	0.2098	34	0.2976
3	0.2243	19	0.2259	35	0.2761
4	0.2334	20	0.1424	36	0.2995
5	0.2051	21	0.1215	37	0.3169
6	0.2183	22	0.2261	38	0.2974
7	0.2037	23	0.2144	39	0.2601
8	0.1861	24	0.2336	40	0.2597
9	0.3733	25	0.2352	41	0.3449
10	0.3782	26	0.1771	42	0.3277
11	0.1961	27	0.1761	43	0.1775
12	0.2018	28	0.2681	44	0.1707
13	0.2021	29	0.2695	45	0.1846
14	0.2188	30	0.2019	46	0.2179
15	0.1789	31	0.2071	47	0.2505
16	0.2021	32	0.2575	48	0.2485

Table S6 The contents of the heavy metal and pesticide residues on the 48 batches of *P. grandiflorum* samples.

Sample no	Heavy metal (mg/kg)					Pesticide residues (mg/kg)		
	Pb	Cd	As	Hg	Cu	BHC	DDT	PCNB

1	-	0.0199	0.0816	0.0118	4.8726	-	-	-
2	0.0871	0.0013	0.0845	-	6.3793	-	-	-
3	0.0710	0.0045	0.0840	0.0512	7.8817	0.0022		-
4	-	0.0089	0.0856	-	7.7753	-	0.0013	-
5	0.0445	0.0021	0.0855	-	8.2778	-	-	-
6	0.0887	0.0159	0.1447	0.0058	4.5787	-	-	-
7	-	0.0189	0.0960	0.0022	8.1489	-	-	-
8	-	-	0.2023	-	4.4993	0.0074	-	-
9	0.0567	0.0011	0.2114	0.0047	5.3973	-	-	-
10	-	0.0049	0.0761	-	4.8166	-	0.0011	-
11	0.0788	-	0.0875	-	4.8766	0.0069	-	-
12	0.0945	-	0.0829	0.0108	6.3796	-	-	-
13	-	-	0.0853	-	7.8616	-	-	-
14	-	0.0061	0.0851	0.0319	7.6756	-	-	-
15	0.0890		0.0862	0.0218	8.1676	-	-	-
16	-	0.0019	0.0856	0.0152	6.5667	0.0078	-	-
17	-	-	0.0844	0.0112	8.1569	-	-	-
18	-	0.0287	0.1335	0.0352	4.6953	-	-	-
19	0.0678	-	0.1640	0.0039	5.3573	0.0051	0.0014	-
20	-	-	0.0934	-	4.8155	-	-	-
21	0.0890	0.0278	0.1112	0.0339	4.8111	-	-	-
22	0.0876	0.0191	0.0836	0.0438	5.1661	-	-	-
23	0.0621	-	0.1984	0.0052	6.1215	-	0.0022	-

24	-	0.0121	0.1352	0.0352	6.0215	-	-	-
25	0.0435	-	0.0836	0.0455	6.0165	-	-	-
26	0.0545	-	0.0814	0.0217	7.1163	-	-	-
27	0.0944	0.0037	0.1315	0.0158	5.8523	-	-	-
28	0.0856	-	0.1610	0.0057	4.9551	-	-	-
29	0.0348	0.0093	0.0914	0.0157	8.2135	0.0062	-	-
30	0.0866	0.0126	0.1179	0.0078	7.8515	-	-	-
31	-	0.0141	0.0881	-	7.1915	-	-	-
32	0.0734	0.0196	0.0849	-	4.8129	-	-	-
33	0.0453	0.0087	0.0861	0.0369	4.8131	-	-	-
34	-	0.0055	0.0822	0.0058	5.1661	-	0.0018	-
35	0.0537	0.0071	0.2113	0.0062	6.3235	-	-	-
36	0.0890	0.0046	0.1544	0.0162	6.3235	-	-	-
37	-	0.0188	0.1444	0.0228	6.6165	-	-	0.0012
38	0.0809	0.0095	0.0829	-	7.3133	0.0080	-	-
39	0.0529	-	0.2416	0.0279	5.8323	0.0062	-	-
40	0.0799	-	0.2117	0.0297	4.9331		-	-
41	0.0557	0.0177	0.2324	0.0322	8.3135		0.0020	-
42	-	0.0142	0.0997	-	7.8315	0.0043	-	-
43	-	0.0265	0.1540	0.0069	7.1315	-	-	0.0010
44	0.0826	0.0219	0.0905	0.0498	4.8329		-	-
45	0.0961	-	0.0867	0.0077	8.4028		0.0016	-
46	-	0.0277	0.0803	0.0192	8.3390		-	-

47	0.0651	-	0.0814	0.0198	8.0018	-	-	-
48	0.0630	-	0.0822	0.0097	8.3028	-	-	-
